# The inheritance of anthracnose (*Colletotrichum sublineola*) resistance in sorghum differential lines QL3 and IS18760

**DOI:** 10.1038/s41598-021-99994-3

**Published:** 2021-10-15

**Authors:** Hugo E. Cuevas, Clara M. Cruet-Burgos, Louis K. Prom, Joseph E. Knoll, Lauren R. Stutts, Wilfred Vermerris

**Affiliations:** 1 USDA-Agricultural Research Service-Tropical Agriculture Research Station, Mayagüez, Puerto Rico; 2 Department of Biology, University of Puerto Rico-Mayaguez Campus, Mayagüez, Puerto Rico; 3 USDA-Agricultural Research Service-Southern Plains Agriculture Research Center, College Station, TX USA; 4 USDA-Agricultural Research Service, Crop Genetics and Breeding Research, Tifton, GA USA; 5 Graduate Program in Plant Molecular and Cellular Biology, University of Florida, Gainesville, FL USA; 6 Department of Microbiology and Cell Science, UF Genetics Institute, and Florida Center for Renewable Fuels and Chemicals, University of Florida, Gainesville, FL USA

**Keywords:** Plant breeding, Genetic linkage study

## Abstract

Anthracnose caused by the fungal pathogen *C. sublineola* is an economically important constraint on worldwide sorghum production. The most effective strategy to safeguard yield is through the introgression of resistance alleles. This requires elucidation of the genetic basis of the different resistance sources that have been identified. In this study, 223 recombinant inbred lines (RILs) derived from crossing anthracnose-differentials QL3 (96 RILs) and IS18760 (127 RILs) with the common susceptible parent PI609251 were evaluated at four field locations in the United States (Florida, Georgia, Texas, and Puerto Rico) for their anthracnose resistance response. Both RIL populations were highly susceptible to anthracnose in Florida and Georgia, while in Puerto Rico and Texas they were segregating for anthracnose resistance response. A genome scan using a composite linkage map of 982 single nucleotide polymorphisms (SNPs) detected two genomic regions of 4.31 and 0.85 Mb on chromosomes 4 and 8, respectively, that explained 10–27% of the phenotypic variation in Texas and Puerto Rico. In parallel, a subset of 43 RILs that contained 67% of the recombination events were evaluated against anthracnose pathotypes from Arkansas (2), Puerto Rico (2) and Texas (4) in the greenhouse. A genome scan showed that the 7.57 Mb region at the distal end of the short arm of chromosome 5 is associated with the resistance response against the pathotype AMP-048 from Arkansas. Comparative analysis identified the genomic region on chromosome 4 overlaps with an anthracnose resistance locus identified in another anthracnose-differential line, SC414-12E, indicating this genomic region is of interest for introgression in susceptible sorghum germplasm. Candidate gene analysis for the resistance locus on chromosome 5 identified an *R*-gene cluster that has high similarity to another *R*-gene cluster associated with anthracnose resistance on chromosome 9.

## Introduction

Sorghum [*Sorghum bicolor* L. (Moench)] is a C4 tropical grass used for food, animal feed, forage, and bioenergy that is drought-tolerant and has lower nutrient requirements compared to many other grasses^1^. Sorghum is the fifth most produced cereal crop behind, wheat (*Triticum aestivium* L.), maize (*Zea mays* L.), rice (*Oryza sativa* L.) and barley (*Hordeum vulgare* L.)^2^. Sorghum production is currently concentrated in the semi-arid regions of the world, where its yield is superior to most other cereal crops. Sorghum’s resilience and adaptability to other environments have led to an expansion of its production to tropical, sub-tropical and temperate regions of the world. The advantages of sorghum as biofuel feedstock are expected to further increase the area of sorghum cultivation^3^. The establishment of sorghum production outside of its natural dry environment presents a challenge due to the presence of multiple abiotic and biotic constraints that can reduce biomass and seed yield and quality.

Anthracnose is a fungal disease that affects all above-ground tissues of sorghum, causing yield losses of both grain and biomass of up to 50% in highly susceptible cultivars^4^. This disease is caused by *Colletotrichum sublineola* P. Henn., in Kabat and Bubák, and the infection results in the formation of an initial lesion on the leaf that has dark red or tan margins and becomes necrotic [reviewed by Stutts and Vermerris^5^]. With time this necrotrophic phase spreads across the plant and the symptoms can be observed on leaves, stalk, peduncle, panicle and seeds^6^. The incidence of anthracnose disease is associated with the warm and humid conditions present in sub-tropical climates^7^, where sorghum production has increased during the last decade^8^. Crop rotation and the application of fungicides are the most common practices to control anthracnose, but the use of fungicides increases production cost^9,10^. The cultivation of anthracnose-resistant cultivars and hybrids is the most effective option to control the disease, but a relatively small number of anthracnose-resistant genotypes with commercial relevance are currently available.

There is a need to identify multiple sources of anthracnose resistance to develop germplasm able to withstand the pathogen’s genetic variation. Multiple resistant accessions have been identified in NPGS tropical germplasm^11–19^, the sweet sorghum collection^20^ and temperate adapted germplasm^21–23^. However, most of these accessions are not being utilized in breeding programs. The inheritance of the resistance loci and genetic relatedness among most of these accessions is unknown, hindering the identification of a subset that contains several resistance sources. In fact, based on genome-wide association analysis (GWAS) using tropical and temperate germplasm, several accessions were demonstrated to share resistance genes (i.e. identical-by-descent), suggesting the presence of few resistance sources among resistant germplasm^19,21^. Therefore, elucidation of the anthracnose resistance mechanism among different resistant sorghum lines is necessary to make optimal use of different resistance sources in sorghum breeding programs.

Inheritance studies and genome-wide association analysis for anthracnose resistance response in field assessment led to the identification of different resistance loci. The resistance response observed in sorghum lines BS04/05, Bk7 and SC155-14E identified anthracnose resistance loci on chromosome 9^24–26^. The resistance loci detected in both Bk7 and SC155-14E were located at the distal end of the short arm of chromosome 9 and may represent the same source of resistance. Two loci located on the long and short arm of chromosome 9 (*Cs1A* and *Cs2A*, respectively) were associated with the resistance response of BS04/05. Three GWAS using NPGS tropical germplasm from Ethiopia, Sudan and the sorghum association panel (SAP) identified candidate genes for loci on chromosome 5 and the distal region of chromosome 9^19,21,27^. Despite the fact that these association studies only explained a limited portion of the observed phenotypic variation, they highlight that these loci are the most common resistance sources present in temperate and tropical germplasm. Therefore, to uncover additional sources of anthracnose resistance, it is necessary to identify and select genetically diverse germplasm that is likely to harbor resistance alleles present at low frequency in temperate and tropical germplasm.

The virulence of *C. sublineola* is not associated with the genetic relatedness among isolates, but is instead defined by its capacity to infect a set of 18 diverse sorghum lines referred to as anthracnose-differentials^28,29^. These 18 anthracnose-differential lines were selected to represent a range in disease resistance mechanisms effective against different pathotypes of *C. sublineola*. In order for sorghum breeding programs to benefit from these different sources of anthracnose resistance, it is necessary to first elucidate the inheritance of the resistance loci. Understanding the pathogen-plant interaction of these anthracnose-differential lines could contribute toward elucidating the diverse molecular mechanisms involved in the resistance response. The use of mapping studies based on phenotypic observations in multiple field locations (with a mix of different pathotypes) combined with greenhouse evaluations with individual pathotypes can provide a better understanding of the resistance mechanism.

Inheritance studies of three anthracnose-differential lines under field conditions (SC748-5, SC112-14 and SC414-12E) identified three major loci located at the distal region of chromosome 5^26,30,31^, and comparative analysis determined that each line contained independent resistance loci. The resistance locus of SC112-14 was fine mapped to a 34-kb genomic region harboring five genes involved in plant immune resistance response instead of pathogen pattern recognition.

Nevertheless, most of the eighteen sorghum-differential lines showed variable anthracnose-resistance responses when challenged by individual pathotypes in the greenhouse versus mixed pathotypes in the field^32^. For instance, in greenhouse evaluations, the lines SC414-12E and SC112-14 were susceptible against some *C. sublineola* isolates from Georgia and Texas^28^. However, both lines showed a broader resistance response across locations during field evaluations^26,31^. In contrast, RTx2536 was shown to be susceptible to anthracnose in the field, but showed resistance against some isolates in the greenhouse. Hence, the anthracnose resistance response in the greenhouse may be limited to the detection of specific molecules or molecular patterns produced by certain isolates, while in the field the resistance mechanism is more complex because it involves the simultaneous recognition of multiple pathotypes and the activation of the entire plant immune system. Given these observations, evaluations of differential lines with select pathotypes in the greenhouse and with a mix of pathotypes in the field are necessary to fully elucidate host–pathogen interactions in sorghum anthracnose disease.

In the current study, the anthracnose-differential lines QL3 and IS18760, which displayed resistance to 18 and 13 pathotypes, respectively, from Texas, Arkansas, Georgia, and Puerto Rico, were crossed with the susceptible line PI609251 to generate a total of 223 recombinant inbred lines (RILs). These RILs were studied in parallel to: (1) determine their resistance responses in field assessments at four locations; (2) identify resistance loci effective under these field conditions based on high-density linkage maps; (3) evaluate the resistance response against eight different *C. sublineola* pathotypes in the greenhouse; (4) identify resistance loci effective against these select *C. sublineola* pathotypes.

## Results

### Anthracnose resistance response in RIL populations

Segregation for the anthracnose resistance response was observed within the RIL populations in the field in Puerto Rico and Texas, while all individual lines of both RIL populations were susceptible in Florida and Georgia. Hence, subsequent analyses were limited to the phenotypic variation observed in Texas and Puerto Rico (Table 1 and Supplementary Table S1). The parental lines QL3 and IS18760 exhibited lower anthracnose infection than the common susceptible parent (PI609251) at either location. Line QL3 exhibited a stronger resistance response against the pathogen population from Puerto Rico than the one present in Texas. In contrast, line IS18760 exhibited a stronger resistance against the pathogen population from Texas than the one present in Puerto Rico. The combined analysis across years identified differences among RILs, and interactions between RILs and year in both populations. The analysis per location revealed differences among RILs in both populations, while a statistically significant interaction between RILs and year was observed in Texas. We observed that of the 223 RILs evaluated at both locations five and eleven RILs were transgressive segregants exhibiting a greater resistance response than QL3 (≤ 2.60) and IS18760 (≤ 2.71), respectively. The broad-sense heritability estimates for anthracnose resistance response in the QL3 and IS18760 populations based on the combined analysis across years were 0.62 and 0.80, respectively. Broad-sense heritability estimates for QL3 and IS18760 were lower in Puerto Rico (0.47 and 0.61, respectively) than those obtained in Texas (0.55 and 0.71, respectively).

**Table 1:** Analysis of variance for the anthracnose resistance response (1–5 scale) of two recombinant inbred line (RIL) populations derived from the crosses between QL3 and IS18760 with a common susceptible line PI609251 evaluated at Puerto Rico (PR) and Texas (TX) in 2016, 2017 and 2018.

Source	QL3 × PI609251						IS18760 × PI609251					
	PR & TX		PR		TX		PR & TX		PR		TX	
	df	*P* value	df	*P *value	df	*P* value	df	*P* value	df	*P* value	df	*P* value
Year	3	*	1	n.s	1	n.s	4	**	1	n.s	2	*
Block(Year)	4	***	2	n.s	2	***	5	***	2	**	3	***
RIL	94	***	92	**	87	**	126	***	123	***	126	***
RIL x Year	227	***	80	n.s	62	***	415	**	92	n.s	200	***

	Means ± standard deviations						Means ± standard deviations					
P_1_	2.60 ± 0.70 a		2.00 ± 0.00 a		2.83 ± 0.75 a		2.71 ± 0.61 a		3.00 ± 0.82 a		2.60 ± 0.52 a	
P_2_	4.00 ± 0.85 b		4.83 ± 0.41 b		3.67 ± 0.77 b		4.00 ± 0.85 b		4.83 ± 0.41 b		3.67 ± 0.77 b	
RILs	3.58 ± 0.60		3.97 ± 0.69		3.17 ± 0.69		3.64 ± 0.57		3.77 ± 0.66		3.58 ± 0.60	
*H*^2^ ± S.E	0.62 ± 0.12		0.47 ± 0.16		0.55 ± 0.15		0.80 ± 0.11		0.61 ± 0.13		0.71 ± 0.12	

n.s., *, ** and *** refers to no significance and significant differences at *P* < 0.05, 0.01 and 0.001, respectively.

*H*^2^ and S.E refers to broad-sense heritability estimates and standard error, respectively.

P_1_ refers to the resistant parent of the RILs population and P_2_ to PI609251.

### High-density linkage maps

A composite linkage map (982 SNPs and 205 RILs) was constructed that contained 941 recombination bins with a combined length of 1,659 cM (Supplementary Table S2). The genome-wide recombination rate was 2.50 cM/Mb with an average distance of 1.67 cM between SNPs (Table 2). Despite the smaller size of the QL3 population, its genetic map contains a greater number of recombination bins than the IS18760 population (851 vs. 745, respectively). These maps of 1,948 and 1,280 cM had genome-wide recombination rates of 2.97 and 1.95 cM/Mb, respectively (Supplementary Table S3 and S4). Based on these average genome-wide recombination rates, the resolution of our maps enables the identification of major loci. Indeed, the SNP ordering based on recombination events was collinear with the BTx623 reference genome, with centromeric regions having most of the SNPs with segregation distortion (Supplementary Fig. S1).

**Table 2:** Comparison of genetic linkage maps constructed with two recombinant inbred line (RIL) populations derived from the crosses between QL3 and IS18760 with a common parental line PI609251, and a composite linkage map for both populations and a subset of RILs selected based on recombination events (i.e. bin map).

Chr	Total SNPs^1^	Tags SNPs^2^	QL3 (n = 96)		IS18760 (n = 109)		Composite (n = 205)		Composite (n = 43)	
			Bin No	Length (cM)	Bin No	Length (cM)	Bin No	Length (cM)	Bin No	Length (cM)
1	430	129	115	228.84	106	190.41	124	219.04	78	216.95
2	235	64	54	180.40	49	96.54	59	138.24	41	160.79
3	388	167	140	282.71	114	170.09	154	226.01	99	250.22
4	275	113	104	255.74	92	168.26	113	227.37	69	205.76
5	278	92	87	233.33	77	123.55	92	182.85	68	243.52
6	574	145	105	145.71	101	125.22	134	137.60	69	146.40
7	142	52	52	129.46	39	64.51	52	96.59	38	142.14
8	168	78	72	169.01	65	132.55	78	164.78	60	201.17
9	214	80	71	172.57	57	97.85	76	135.84	49	154.47
10	131	62	51	150.23	45	110.74	59	130.94	42	146.87
Total	2835	982	851	1948.00	745	1279.73	941	1659.25	633	1868.00

^1^Single nucleotide polymorphisms identified by the genotype-by-sequencing analysis of the RIL populations.

^2^Single nucleotide polymorphisms selected for the construction of genetic linkage maps.

### QTL mapping of anthracnose resistance response

**Figure 1:**
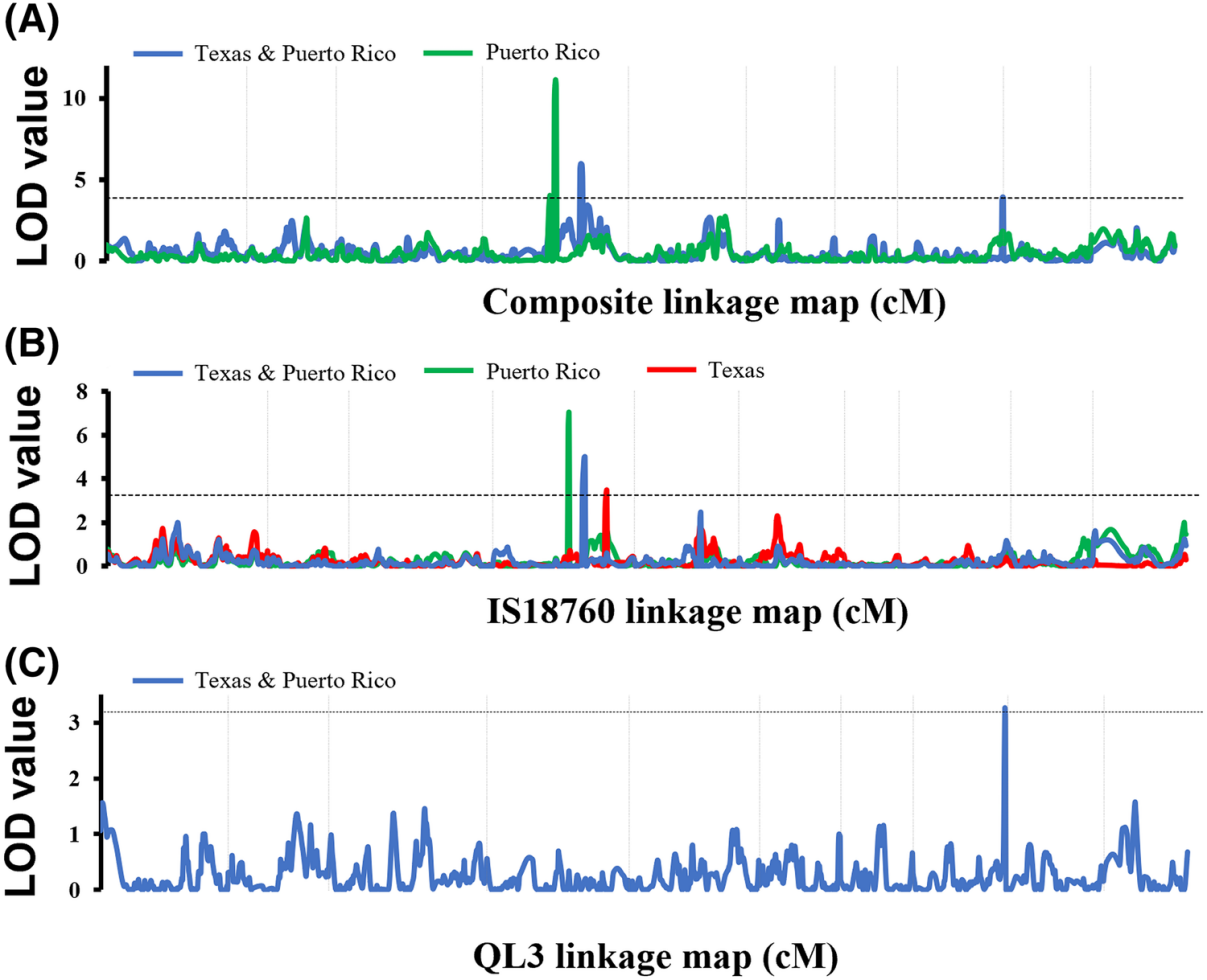
Genome scan for anthracnose resistance response of recombinant inbred lines (RILs) derived from the crosses between IS18760 and QL3 with the common parental line PI609251. (**A**) Joint inclusive composite interval mapping (JICIM) using a composite linkage map and the anthracnose resistance response observed in Puerto Rico and across Texas and Puerto Rico. (**B**) JICIM using the linkage map derived from IS18760 RIL population and anthracnose resistance response observed in Puerto Rico, Texas and across both locations. (**C**) JICIM using the linkage map derived from QL3 RIL populations and anthracnose resistance response observed across Texas and Puerto Rico. Horizontal dashed lines mark the significance threshold (*P* < 0.05), and vertical dashed lines delimit the chromosomes.

Using joint inclusive composite interval mapping (JICIM) two genomic regions were detected on chromosomes 4 and 8 that explained 10–27% of the variance in anthracnose resistance observed in Puerto Rico and across locations (Table 3). A genome scan based on the resistance response in Puerto Rico identified a 1.6 Mb genomic region on chromosome 4 (52.42–54.05 Mb) containing two segments that individually explain 9.5 and 15% of the variance (Fig. 1). Similarly, a genome scan based on resistance response across locations identified 1.03 Mb and 0.85 Mb genomic regions on chromosomes 4 (55.77–56.73 Mb) and 8 (61.64–62.49 Mb), respectively, which explained 6.5 and 4.0% of the variance, respectively. The limited number of recombination events within this 4.31 Mb genomic region on chromosome 4 (52.42–56.73 Mb) resulted in it being considered as a single QTL (*qSbCs04.52-57*). The resistance allele in the QTL in chromosome 8 (*qSbCs08.61-63*) was inherited from PI609251.

**Table 3:** Genomic regions associated with the anthracnose resistance response revealed by the QTL analysis of two sets of recombinant inbred lines derived from the crosses between QL3 and IS18760 with a common parental line PI609251 evaluated at Puerto Rico and Texas in 2016, 2017 and 2018.

Linkage Map	Location	Chr	Region (Mbp)	LOD	P.V.E.^2^	Additive effects^1^		QTL name
						IS18760	QL3	
Composite^3^	Puerto Rico	4	52.42–53.32	4.03	9.47	− 0.01	0.27	*qSbCs04.52-57*
		4	53.82–54.05	11.13	15.06	− 0.29	− 0.31	
	Texas & Puerto Rico	4	55.77–56.73	5.98	6.45	− 0.25	− 0.08	*qSbCs04.52-57*
		8	61.64–62.49	3.91	3.95	0.10	0.20	*qSbCs08.61-63*
IS18760^4^	Puerto Rico	4	54.41–54.70	6.99	26.82	− 0.32	*n.a*	*qSbCs04.52-57*
	Texas	4	60.47–61.18	3.46	14.41	− 0.22	*n.a*	*qSbCs04.60-62*
	Texas & Puerto Rico	4	55.77–56.84	5.00	21.06	− 0.24	*n.a*	*qSbCs04.52-57*
QL3^4^	Texas & Puerto Rico	8	61.64–62.49	3.27	15.07	*n.a*	0.22	*qSbCs08.61-63*

^1^Negative sign indicates the resistant allele is derived from resistant parent (IS18760 or QL3).

^2^Percent of variance explained by the QTL.

^3^LOD values based on joint inclusive composite interval mapping as implemented in QTL IciMapping using a 982 SNPs composite linkage map.

^4^LOD values based on inclusive composite interval mapping as implemented in QTL IciMapping using 982 SNPs linkage map from each recombinant inbred line population.

The inclusive composite interval mapping (ICIM) based on each RIL population provided further insights in the QTLs on chromosomes 4 and 8 (Table 3). A genome scan using the IS18760 genetic linkage map detected three adjoining regions on chromosome 4 for anthracnose resistance response observed in Puerto Rico (54.41–54.70 Mb), Texas (60.47–61.18 Mb) and across locations (55.77–56.84 Mb) that explained up to 27% of the observed variance. The genomic regions identified with the resistance response in Puerto Rico and across locations overlap with previous QTLs detected by JICIM (*qSbCs04.52-57*). However, the genomic region associated with the resistance response in Texas is located 3.47 Mb upstream, thus was considered as a separate QTL (*qSbCs04.60-62).* The genome scan using the QL3 map detected a region on chromosome 8 that explains 15% of the variance across locations and overlaps with the QTL identified by JICIM (*qSbCs08.61-63*).

### Genome mapping of resistance response against eight anthracnose pathotypes

Two subsets of 21 and 22 RILs from IS18760 and QL3 populations that contained 67% of the recombination events were selected and evaluated against eight pathotypes: four from Texas, and two each from Arkansas and Puerto Rico. The screening of these 43 RILs identified segregation for anthracnose resistance response when they were challenged against one particular *C. sublineola* pathotype (Table 4). A total of five RILs from the QL3 population were resistant (≤ 2 on a 1–5 scale; absence of acervuli on leaves) against all eight pathotypes. In contrast, none of the RILs were completely susceptible to all eight pathotypes. The larger number of susceptible RILs were observed with pathotype 20 (22 RILs) and pathotype 31 (21 RILs) from Texas. Moreover, we observed that a greater number of RILs derived from IS18760 were susceptible to these pathotypes compared to RILs from the QL3 population. The segregation pattern of these 43 RILs against each pathotype was different, suggesting the presence of multiple resistance loci.

**Table 4:** Anthracnose resistance response of 43 recombinant inbred lines (RILs) derived from the crosses between QL3 and IS18760 (IS) with a common parental line PI609251 (P_2_) evaluated against eight pathotypes from Arkansas (AK), Puerto Rico (PR) and Texas (TX), USA.

RILs	AK		PR		TX				RILs	AK		PR		TX			
	AMP-048	AMP-050	Path. 32	Path. 36	Path. 20	Path. 26	Path. 29	Path. 31		AMP-048	AMP-050	Path. 32	Path. 36	Path. 20	Path. 26	Path. 29	Path. 31
QL3 (P_1_)	R	R	R	R	R	R	R	R	IS (P_1_)	R	R	R	R	R	R	R	R
QL3-005	R	R	S	R	S	R	R	S	IS-005	R	R	R	R	S	R	R	S
QL3-008	R	R	R	R	R	R	S	R	IS-034	S	S	S	R	S	S	S	S
QL3-013	S	S	S	S	S	S	R	R	IS-036	R	S	R	R	S	S	R	S
QL3-033	R	R	R	R	R	R	R	R	IS-039	S	S	S	R	S	S	R	R
QL3-034	R	S	S	S	S	S	R	S	IS-045	R	R	R	S	R	R	R	R
QL3-035	R	R	S	S	S	R	S	S	IS-048	S	R	R	R	R	R	R	R
QL3-038	R	R	R	R	R	R	R	R	IS-058	R	R	R	S	S	S	S	S
QL3-039	S	R	S	S	S	R	R	S	IS-059	R	R	R	R	R	R	S	R
QL3-040	R	R	R	R	R	R	R	R	IS-060	S	R	R	S	R	R	S	S
QL3-041	R	R	R	R	S	R	R	R	IS-065	R	R	R	R	R	S	R	S
QL3-045	R	R	R	R	R	R	R	R	IS-068	R	R	R	R	S	R	R	R
QL3-046	R	R	R	R	R	R	R	R	IS-071	R	S	R	R	R	S	S	S
QL3-051	S	S	R	S	S	R	R	S	IS-073	R	S	S	S	S	S	R	S
QL3-056	R	R	S	R	S	R	S	S	IS-079	R	S	S	S	S	R	S	S
QL3-062	R	S	S	S	S	S	R	S	IS-091	S	S	S	S	S	R	S	S
QL3-070	R	S	R	S	S	R	R	R	IS-096	R	S	S	S	S	S	S	S
QL3-073	R	R	S	R	R	R	R	S	IS-105	R	R	S	R	R	S	R	S
QL3-075	R	R	R	S	R	R	S	R	IS-118	S	S	S	R	S	S	R	R
QL3-086	R	R	R	R	R	S	S	R	IS-123	R	S	S	R	R	S	R	R
QL3-091	R	S	R	R	R	R	R	R	IS-125	R	S	R	R	R	R	R	R
QL3-095	R	S	R	R	R	R	R	R	IS-144	S	S	S	R	S	S	S	S
QL3-109	S	R	R	R	R	S	R	R	P_2_	S	S	S	S	S	S	S	S

R and S refers to resistant and susceptible, respectively.

The *C. sublineola* pathotypes were based on the classification described by Prom et al.^13,28^.

**Figure 2:**
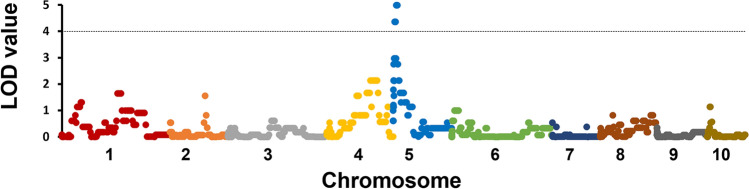
Manhattan plot for the single marker analysis of 982 SNPs and the anthracnose resistance response of a representative subset (i.e. BIN Map) of 21 recombinant inbred lines (RILs) derived from the crosses between IS18760 and PI609251 evaluated in the greenhouse against the pathotype AMP-048 from Arkansas, U.S.A. Horizontal dashed line marks significance threshold based on 1000 permutations (*P* < 0.05).

A genome scan for anthracnose resistance response against the eight pathotypes in the 43 RILs did not reveal any significant associations with a particular genomic region. However, a genome scan limited to the 21 RILs from IS18760 identified an association between genetic variation at the telomeric region of the short arm of chromosome 5 and resistance against pathotype AMP-048 from Arkansas (Fig. 2). This 7.57 Mb genomic region (8.22–15.79 Mb) constitutes of three bins containing 303 putative genes, including nineteen (*Sobic.05G070500, Sobic.05G071700, Sobic.05G071900, Sobic.05G072000, Sobic.05G075100, Sobic.05G075600, Sobic.05G075800, Sobic.05G076100*, *Sobic.05G076200*, *Sobic.05G076301*, *Sobic.05G076400*, *Sobic05G076500, Sobic.05G091300, Sobic.05G092600, Sobic.05G094001, Sobic.05G95000, Sobic.05G095600, Sobic.05G095700* and *Sobic.05G096300*) that have features shared by *R*-genes. Remarkably, these *R-*genes were distributed among the three bins with the strongest association in the third bin [Chr5: 12.02–15.79; LOD value = 4.99].

## Discussion

We studied the resistance response of anthracnose-differential lines QL3 and IS18760 at four field locations and against eight *C. sublineola* pathotypes from Texas, Arkansas and Puerto Rico. The results confirmed that anthracnose resistance response depend on the *C. sublineola* population present at each location. Multiple studies have shown that anthracnose resistance responses observed in the greenhouse failed under field conditions^28,31,33^. Small changes in the environmental conditions in the field can favor a greater anthracnose disease pressure to which the plant can respond through a combination of pathogen recognition, activation of the plant immune system and activation of specific metabolic pathways that lead to the production of defense compounds. In contrast, the uniform conditions in the greenhouse (e.g. consistent soil type and temperature, regular watering, absence of other pathogens, etc.) are the most suitable for detailed studies of plant-pathogen interactions. The combination of two approaches provided different insights into the resistance mechanism that can ultimately help in the development of improved sorghum germplasm with a broad resistance response.

The large diversity of *C. sublineola* pathotypes within populations in the field causes a large variation in the resistance response among different sorghum genotypes^28^. Inheritance studies of anthracnose resistance response based on field conditions have identified genomic regions containing *R*-genes, transcription factors and defense-related proteins, suggesting the interaction of multiple defense mechanisms^25,26,30,31^. If multiple *R*-genes are involved in the defense against different field pathotypes, it is not possible to detect these genomic regions due to the lack of a clear inheritance pattern in a mapping population. Instead, genomic regions associated with other common downstream factors involved in the signaling cascade will likely be identified as being associated with the resistance response. Candidate gene analysis of the QTL identified genes on chromosomes 4 and 8 that encode transcription factors known to be involved in plant immunity^34^ and other genes encoding proteins containing leucine-rich repeats. Both genomic regions explained only a limited portion of the variance for anthracnose resistance, suggesting their effects are determined by the parallel additive effects of other, yet to be detected loci. A gene expression atlas derived from anthracnose-differential line SC283 identified genes encoding immune receptors, MAPKs, pentatricopeptide repeat proteins, and WRKY transcription factors as the most highly expressed genes in response to infection^35^. Increasing the mapping population size or fine mapping these loci are not affordable approaches to detect minor additive effect genes. However, further association studies based on gene expression analysis (i.e. eQTL)^36^ could lead to the identification of other genomic regions involved in the anthracnose resistance response.

The confirmation of a QTL by independent inheritance studies in different genetic backgrounds is an important step before its use in breeding programs. The QTL on chromosome 4 overlaps with a QTL identified in resistant lines SC155-14E and SC414-12E (Supplementary Fig. S2)^26^. The alignment of *qSbCs04.52-57* and *qSbCs04.60-62* with two QTL detected in SC155-14E and SC-414-12E delimited two common genomic regions of 2.06 (53.95–56.01 Mb) and 0.21 Mb (60.47–60.68 Mb). This genomic region on chromosome 4 has a minor effect in the genetic backgrounds of SC155-14E and SC-414-12E, because the resistance response is controlled by major QTLs on chromosomes 9 and 5, respectively. Hence, the effect of this genomic region is determined by the genetic background and its interaction with other major resistance loci. Due to the absence of another major resistance locus in IS18760, the additive effects of this genomic region are much more important for anthracnose resistance in this line.

It has been documented (in rice) that the resistance response mechanism against one particular pathotype may differ from the resistance response under field conditions in the presence of multiple pathotypes^37^. In this study we identified three bins of 0.86, 2.03 and 4.68 Mb on chromosome 5 that were effective against pathotype AMP-048 from Arkansas. The 2.03 Mb region contains eight *R*-genes and the amino acid sequence similarity among the proteins encoded by six of these genes (*Sobic05G075100*, *Sobic05G075600*, *Sobic05G075800*, *Sobic05G076100*, *Sobic05G076200*, *Sobic05G076400*) is greater than 70%. Remarkably, the proteins encoded by this cluster of six *R*-genes also have amino acid sequence similarity (> 70%) with proteins encoded by another *R*-gene cluster at the distal end of chromosome 9 (*Sobic09G013000*, *Sobic09G013100* and *Sobic09G013300*), which has been previously associated with anthracnose resistance response^19,25^. Moreover, the resistance response of anthracnose-differential line SC112-14 against AMP-048 could not be associated to either genomic region, while the resistance response against the other seven pathotypes was determined by a 34 kb genomic region on chromosome 5^31^. The interaction among effectors produced by AMP-048 and proteins encoded by some of the genes in this *R*-gene cluster might be associated with the activation of the plant immune system, while resistance alleles for this *R*-gene cluster are absent in SC112-14. Therefore, combining the genomic regions of SC112-14 and IS18760 may be effective at producing a broader resistance response.

The lack of association between the genetic variation of RILs from QL3 and IS18760 and the resistance response against seven pathotypes suggested the resistance mechanism for these pathotypes might involve the interaction of multiple genes. Indeed, a single pathotype produces dozens of elicitors and effectors that can be recognized directly or indirectly by cell surface receptors and R proteins^38^, which initiates a signaling cascade leading to a defense response^39^. Understanding this plant-pathogen interaction is crucial for the establishment of signaling pathways that regulate the plant defense response. A dual gene expression analysis of both host (*Nicotiana benthaminana*) and pathogen (*Phytophtora palmivora*) has been used successfully to identify conserved effectors^40^. Indeed, future dual transcriptome profiling of this subset of sorghum RILs together with the eight isolates may lead to the identification of resistance genes associated with the recognition of multiple *C. sublineola* elicitors and effectors.

The anthracnose resistance response in sorghum relied on the pathogen diversity present in the trial. Anthracnose-differential line QL3 was resistant against 18 pathotypes in the greenhouse screening^28^ However, it was susceptible or moderately resistant in field screenings. In contrast, other anthracnose-differential lines (e.g. SC112-14, SC414-12E) that showed susceptibility to some pathotypes were highly resistant in the field^26,31^. The moderate resistance of anthracnose-differential line QL3 under field conditions might be determined by the simultaneous infection by multiple pathotypes. In fact, most of the field resistance response is thought to involve multiple QTL acting consecutively at different times during the pathogen infection cycle or through plant development^41^. Hence, the resistance response of anthracnose-differentials QL3 and IS18760 is likely controlled by multiple minor QTLs, two of which were identified on chromosomes 4 and 5. Most likely, the resistance response of most anthracnose-differentials is controlled by the synergistic effects of multiple minor QTLs. Even though this may limit their utility in sorghum breeding programs, these anthracnose-differentials remain valuable to unravel molecular mechanisms underlying the anthracnose resistance response.

## Conclusion

Anthracnose resistance responses in anthracnose-differential lines IS18760 and QL3 were not effective under field conditions in Florida and Georgia, and were moderately effective in the field conditions in Puerto Rico and Texas. Two QTLs on chromosomes 4 and 8 were associated with this field resistance response, while a resistance locus on chromosome 5 was associated with the resistance response against one *C. sublineola* pathotype from Arkansas in a greenhouse study. Candidate gene analysis identified an *R*-gene cluster in a locus on chromosome 5 that displays sequence similarity with another *R*-gene cluster on chromosome 9 previously associated with anthracnose resistance response. Likewise, the locus on chromosome 4 validated QTLs identified in resistant lines SC155-14E and SC414-12E, indicating this genomic region can be introgressed into susceptible germplasm to provide anthracnose resistance.

## Materials and methods

### RILs and field anthracnose severity

Two sets of recombinant inbred lines (RILs; F_5:6_) were obtained by using the single-seed-descent method from the cross of anthracnose-differential lines QL3 and IS18760 with a common susceptible line PI609251. These two anthracnose-differential lines are originally from India and Sudan, respectively, while the susceptible common parental line is originally from Mali. These populations, QL3 × PI609251 and IS18760 × PI609251, are referenced according to their unique parents (i.e. QL3 and IS18760), and comprise 96 and 127 RILs, respectively.

The 223 RILs, parental lines and control checks [SC748-5 (resistant) and BTx623 (susceptible)] were planted during two years (2016 and 2017) at the research farm of the USDA-ARS Tropical Agriculture Research Station in Isabela, Puerto Rico, U.S.A (18° 0.28′ 18.4″ N, 67° 02′ 37.2″ W) and at the Research Farm of the Texas A&M University at College Station, Texas, U.S.A. (30° 31′ 55.5″ N, 96° 25′ 23.7″ W). The IS18760 population was planted for a third year (2018) at College Station, Texas. Both populations were also planted at the Black Shank Farm of the University of Georgia in Tifton, Georgia, U.S.A. (31° 29′ 58.9″ N, 83° 32′ 53.8″ W), and at the University of Florida Suwannee Valley Agricultural Research and Education Center near Live Oak, Florida, U.S.A. (30° 18′ 47.8″ N, 82° 54′ 07.8″ W) in 2016. The experimental design at all locations was a randomized complete block design with two replicates, consisting of 3.1 m single row plots and 0.9 m between rows. All the experiments comply with the United States Department of Agriculture, Agricultural Research Services guidelines. We obtained permission to use the sorghum seeds and to collect plant tissue before the experiments.

### Anthracnose evaluation in the fields

Leaf samples with characteristic anthracnose symptoms (presence of acervuli) were collected from each location, and single spore isolates were obtained and identified as previously described^28,42^. To reach a more uniform disease distribution in the field several plants per row were manually inoculated according to Prom, Perumal^42^. Briefly, three to five *C. sublineola* isolates from each location were cultured on half strength potato-dextrose agar, followed by the inoculation and colonization of autoclaved sorghum seeds during a period of 2 weeks. Approximately ten *C. sublineola*-colonized seeds were placed into the leaf whorl of 30–45 day-old-plants (the exact time depended on plant development and varied by genotype). The anthracnose resistance responses of RILs were determined after flowering (hard-dough stage to physiological maturity) using a 1 to 5 scale which has been proven successful for identifying anthracnose resistance loci^19,21,25,31,43^. The whole plot (row) was visually inspected for presence of anthracnose disease and the most severely infected plants were scored as: 1 = no symptoms or chlorotic flecks in the plot; 2 = hypersensitive reaction, but no acervuli present in the plot; 3 = infected bottom leaves with acervuli formation in at least one plant within the plot; 4 = necrotic lesions with acervuli observed on bottom leaves and spreading to middle leaves in at least one plant within the plot; and 5 = most leaves necrotic due to infection, including infection of the flag leaf in at least one plant within the plot. The anthracnose resistance responses of the RILs was categorized as resistant (≤ 2.0 and absence of acervuli on leaf) or susceptible (> 2.0 and presence of acervuli on leaf) based on this 1–5 scale.

### Statistical analysis

The anthracnose resistance response of each RIL population within location and across locations were estimated based on least square means. Locations and years were combined and subjected to analysis of variance using the *proc mixed covtest* method *type 3* procedure of SAS 9.4 (SAS Institute, Cary, NC). The location was considered fixed, whereas years, blocks in years, RILs and the interaction of RILs by years were treated as random effects. The least square means of anthracnose resistance response of each RIL were estimated for both across and within location. The broad-sense heritability (*H*^2^) across and within locations was estimated using the formula:

$$ H^{2} = \frac{{\sigma_{g}^{2} }}{{\sigma_{g}^{2} + \frac{{\sigma_{GxE}^{2} }}{e} + \frac{{\sigma_{e}^{2} }}{re} }}, $$

where $\sigma_{g}^{2}$, $\sigma_{GXE}^{2}$, $\sigma_{e}^{2}$ refer to the genotypic (RILs), genotype-by-environment (RIL x Year), and error variances, respectively, while *e* and *r* are the number of environments (Years) and blocks, respectively^44^. Standard error of *H*^2^ estimates were determined according to Hallauer and Miranda^45^.

### Genotyping-by-sequencing of RILs populations

A leaf bulk tissue from 3 to 5 seedlings of RILs and parental lines were collected and DNA isolated using the method described by Guillemaut and Marechal-Drouard^46^ with some modifications and purified using ZR 96 DNA Clean & Concentrator-5 (Zymo Research, Irvine, CA, USA). Genotype-by-sequencing (GBS) libraries were prepared using the restriction enzyme *Ape*KI for digestion^47^ and sequenced in an Illumina Nova Seq 6000 with a coverage of 2 million reads per RIL at the University of Wisconsin Biotechnology Center DNA Sequencing Facility (University of Wisconsin, Madison, WI). The Tassel 5 GBS v2 Pipeline^48^ was used to process the data and SNP calling was based on the most recent version of the BTx623 sorghum genome (version 3.1; www.phytozome.net, accessed June 26, 2019). The raw genotypes involved 191,463 SNPs for both RILs populations, of which 3,049 SNPs were retained after filtering by minor allele frequency (MAFs) (> 0.40), percent of missing (< 20%) and maximum heterozygous proportion (< 0.15). Subsequently, missing data were imputed using Beagle 4.1^49^, while heterozygous and imputed genotypes with a probability call of < 0.80 were retained as missing data. This new imputed genotype data was filtered for MAFs > 0.40, percent of missing data (< 15%), and segregation distortion against a 1:1 expected ratio [χ^2^ *P*(value) < 0.05], which resulted in a total of 2,843 SNPs.

### High-density linkage maps construction

Linkage maps were built for each RIL population, and a composite map based on the merger of both RIL populations. First, the composite map was constructed using the Linux version of MSTmap software (http://mstmap.org/)^50^ using a LOD criterion > 10, Kosambi mapping distance, and genotyping error detection. The resulting map was visually inspected to identify and remove SNPs with unlikely double recombination events within each bin and similar genotyping information. A total of 982 SNPs were retained and the linkage map was rebuilt using MSTmap software as previously described^50^ (Supplementary Table S2). Subsequently, linkage maps for each RIL population were built using these 982 SNPs and MSTmap software as previously described (referred as IS18760 and QL3 maps, respectively; Supplementary Table S3 and S4). The collinearity of these three linkage maps against BTx623 reference genome [version 3.1; Phytozome 13 (www.phytozome.net), accessed June 2020] was confirmed by plot visualization (Supplementary Figure S1).

### QTL mapping

Joint inclusive composite interval mapping [JICIM;^51^] was conducted using the anthracnose resistance response of both RIL populations across and within location as implemented in QTL IciMapping v4.2^52^. The additive JICIM method was used to scan the composite linkage map with a walking speed of 1 cM. The threshold to determine a statistically significant QTL was calculated with 1,000 permutations for experiment-wise error rates of α = 0.05. In addition, inclusive composite interval mapping [ICIM;^53^] was conducted using the separate anthracnose resistance response of each RIL populations across and within locations as implemented in QTL IciMapping 4.1. The additive ICIM method was used to scan QL3 and IS18760 linkage maps with a walking speed of 1 cM. The threshold to determine a statistically significant QTL was calculated with 1,000 permutations for experiment-wise error rates of α = 0.05. Candidate genes within associated genomic regions were identified based on the most recent annotation of the BTx623 sorghum reference genome [version 3.1; Phytozome 13 (www.phytozome.net) accessed June 2020].

### Genome mapping of resistance response against eight anthracnose pathotypes

Two subsets of 21 and 22 highly informative RILs from IS18760 and QL3 populations, respectively, were selected using its linkage map and Mappop v.1.0 software (https://visionlab.web.unc.edu/software-and-databases/mappop/)^54^. The *SAMPLEMAX* command with a 0.30 fraction ratio was applied to both populations to generate a suitable subset for genome mapping with a minimal loss of precision. A composite map was constructed using these 43 RILs and MSTmap software^50^ using a LOD criterion > 3.0, Kosambi mapping distance, and genotyping error detection.

These 43 RILs, parental lines (IS18760, QL3 and PI609251), and reference lines BTx623 (susceptible), TAM428 (susceptible) and SC748-5 (resistant) were evaluated during the Spring and Fall of 2017 in the greenhouse facilities of the Southern Plains Agriculture Research Center, College Station, Texas, USA. The greenhouse experimental design was a randomized complete block design with two replicates using 49 tall tree pots (11.4 L) per block. A total of four individual plants per genotype were grown in each tree pot and evaluated for anthracnose resistance response.

A total of eight pathotypes that consist of two from Arkansas (AMP-048, and AMP-050), two from Puerto Rico (Pathotypes 32 and 36), and four from Texas (Pathotypes 20, 26, 29 and 31) were separately used in the greenhouse trial (i.e. eight independently experiments of 98 tree pot each one with four plants). These pathotypes were previously genetically characterized and represent most of the genetic diversity of *C. sublineola*^28^. At the 8–10 leaf stage, the plants were inoculated by placing approximately ten *C. sublineola*-colonized sorghum seeds in the whorl and by spraying 3–5 mL of a conidial suspension (10^6^ conidia mL^−1^). To maintain an adequate humid environment for disease development, plants were misted for 30 s at 45-min intervals for 8 h during the length of the experiment. The anthracnose resistance responses of the RILs were determined approximately 35–45 days after inoculations and rated as resistant (≤ 2 on 1–5 scale; absence of acervuli on inoculated leaves) or susceptible (> 2 on 1–5 scale; presence of acervuli on inoculated leaves).

A single-marker analysis was conducted in QTL IciMapping 4.1^52^ using the binary data (*i.e.* resistant or susceptible) to identify anthracnose resistance loci for each pathotype. The analysis was conducted using the separate anthracnose resistance response of each subset of the RIL populations and both subsets at once. The threshold to determine a statistically significant QTL was calculated with 1,000 permutations for an experiment-wise error rate of α = 0.05. Candidate genes within associated genomic regions were identified based on the most recent annotation of the BTx623 sorghum reference genome [version 3.1; Phytozome 13 (www.phytozome.net) accessed June 2020].

### Consent to publication

All the authors consented for publication.

## Supplementary Information

Supplementary Information 1.

Supplementary Information 2.

Supplementary Information 3.

Supplementary Information 4.

## Data Availability

All data is available as Supplementary materials.
