# Supplementary material for: The inheritance of anthracnose (Colletotrichum sublineola) resistance in sorghum differential lines QL3 and IS18760
Source: Sci Rep. 2021 Oct 15;11:20525. doi: 10.1038/s41598-021-99994-3 (PMC8519964; doi:10.1038/s41598-021-99994-3)
Supplement: Supplementary file 1 — Supplementary Information 1. [file 41598_2021_99994_MOESM1_ESM.docx]

**Supplementary Figure S1** Collinearity between the BTx623 sorghum reference genome and the three SNP-based linkage maps constructed from the two RILs populations derived from crossing QL3 and IS18760 with a common parental line PI609251. **A)** Composite linkage map built using both RILs populations; **B)** Linkage map built with IS18760 RILs population; **C)** Linkage map built with QL3 RILs population

**Supplementary Figure S2** Alignment of genomic regions associated with anthracnose resistance response in sorghum lines IS18760, SC414-12E and SC155-14E (Patil et al. 2017).
