# Supplementary figures and images for: The inheritance of anthracnose (Colletotrichum sublineola) resistance in sorghum differential lines QL3 and IS18760
Source: Sci Rep. 2021 Oct 15;11:20525. doi: 10.1038/s41598-021-99994-3 (PMC8519964; doi:10.1038/s41598-021-99994-3)

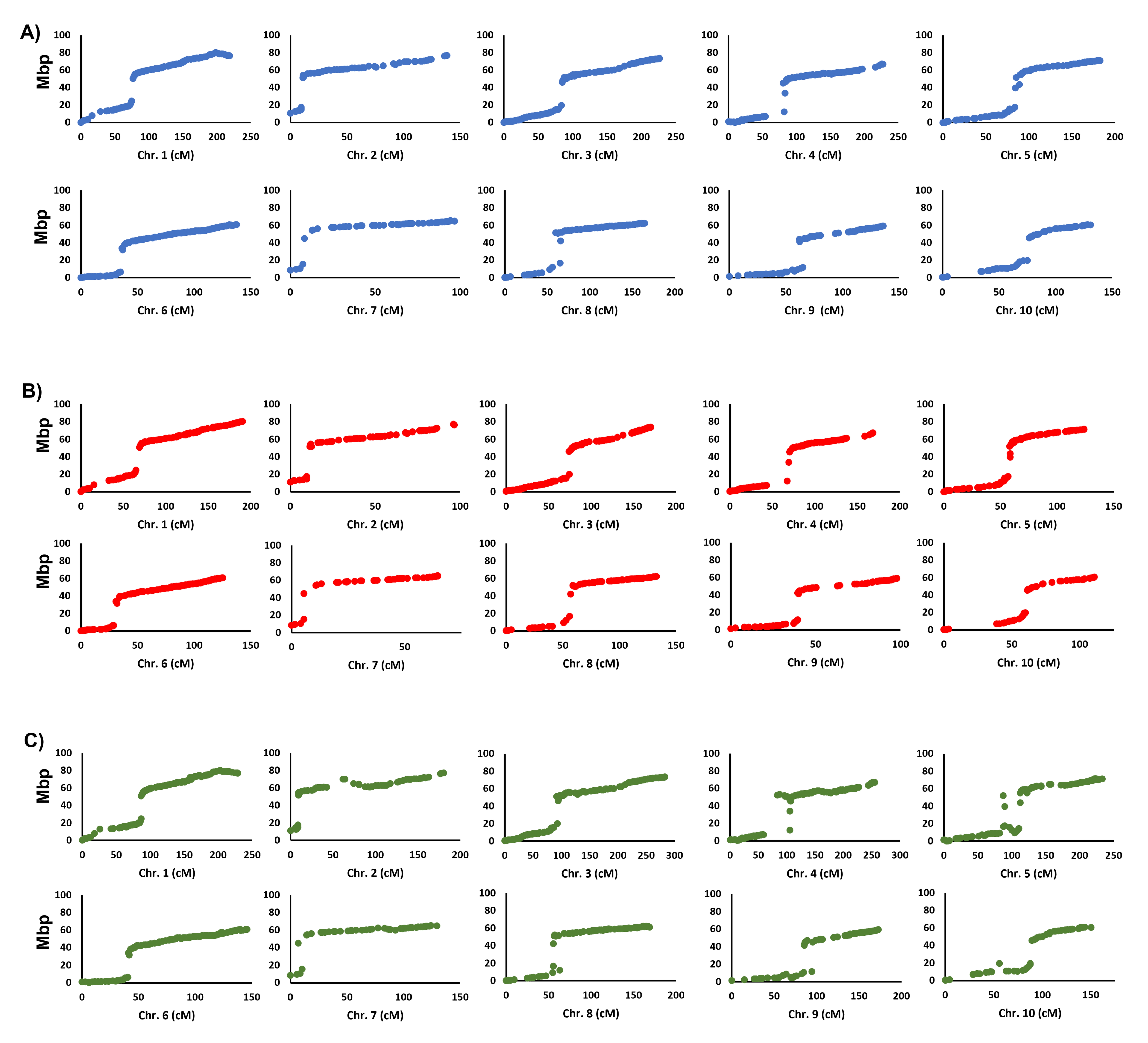

Supplement: Supplementary file 2 — Supplementary Information 2. [file 41598_2021_99994_MOESM2_ESM.jpg]

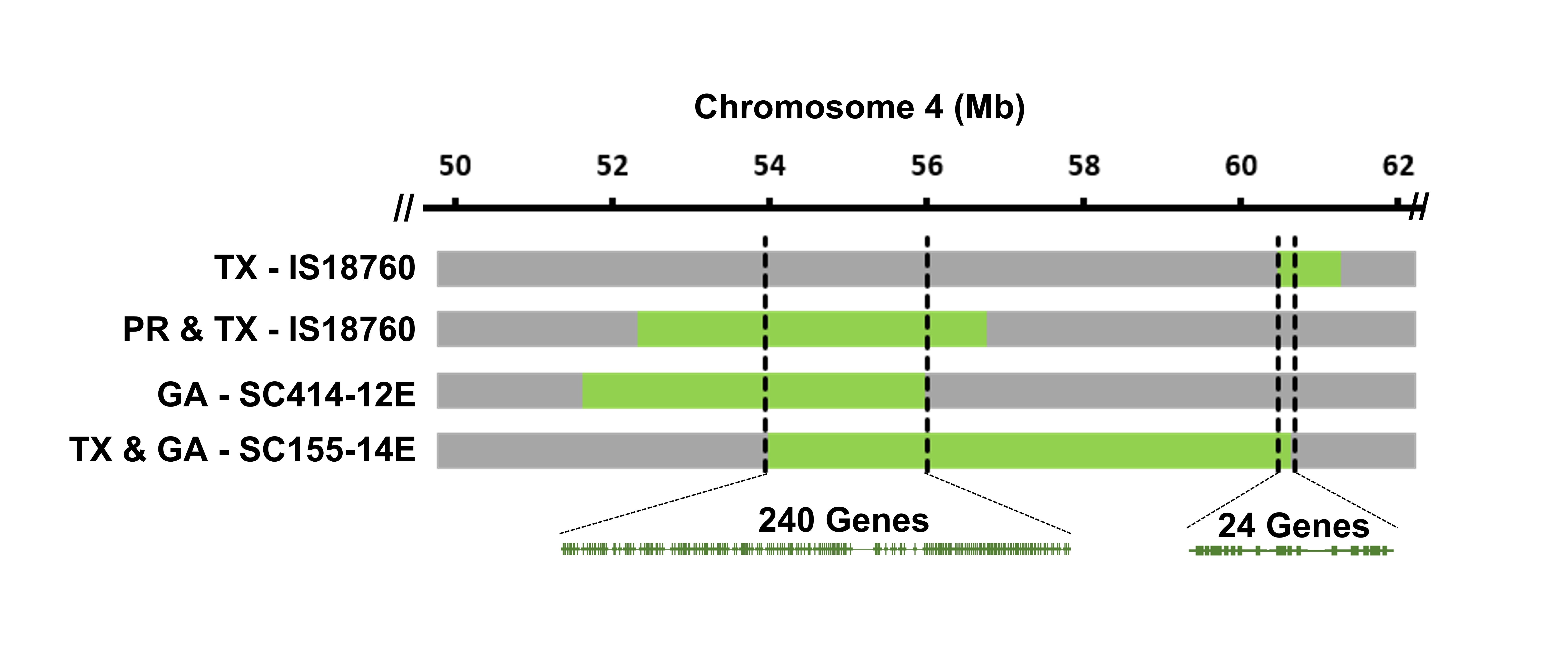

Supplement: Supplementary file 3 — Supplementary Information 3. [file 41598_2021_99994_MOESM3_ESM.jpg]
